# Urinary volatile organic compounds in overweight compared to normal-weight children: results from the Italian I.Family cohort

**DOI:** 10.1038/s41598-017-15957-7

**Published:** 2017-11-15

**Authors:** Rosaria Cozzolino, Beatrice De Giulio, Pasquale Marena, Antonella Martignetti, Kathrin Günther, Fabio Lauria, Paola Russo, Matteo Stocchero, Alfonso Siani

**Affiliations:** 1 Institute of Food Science, CNR, Avellino, Italy; 2 Leibniz Institute for Prevention Research and Epidemiology - BIPS, Bremen, Germany; 3 S-IN Soluzioni Informatiche Srl, Vicenza, Italy

## Abstract

Accumulating evidence shows that urinary volatile organic compounds (VOCs) could be perturbed in many physiological and pathological states, including several diseases and different dietary exposures. Few studies investigated the urinary metabolic signature associated to excess body weight and obesity in adult populations, while a different VOCs profile was found in exhaled breath in obese as compared to lean children. Aim of this study was to evaluate the VOCs profile in the urine of 21 overweight/obese (OW/Ob) and 28 normal-weight (NW) children belonging to the Italian cohort of the I. Family study. Urine samples were analysed by Solid Phase Micro-Extraction (SPME) GC-MS under both acidic and alkaline conditions, in order to profile a wider range of urinary volatiles with different physicochemical properties. Multivariate statistics techniques were applied to bioanalytical data to visualize clusters of cases and detect the VOCs able to differentiate OW/Ob from NW children. Under alkaline conditions, fourteen VOCs were identified, distinguishing OW/Ob from NW children. Our results suggest that VOCs signatures differ between OW/Ob and NW children. However, the biological and pathophysiological meaning of the observed differences needs to be elucidated, in order to better understand the potential of urinary VOCs as early metabolic biomarkers of obesity.

## Introduction

Childhood obesity incidence has globally risen at an alarming rate during last decades. Although the increasing rates are apparently plateauing in some Western countries, it remains a serious challenge and a public health priority, given that obesity during childhood is associated with an increased risk of morbidity and mortality due to non-communicable diseases in adulthood^1^.

The long-term metabolic consequences of childhood obesity are mainly associated with an excessive accumulation of body fat, which in turn leads to an increased risk to develop non-communicable diseases, like type 2 diabetes mellitus and cardiovascular diseases^2^. However, the mechanistic pathways by which adiposity may induce metabolic perturbations are not fully understood^3^. The identification in the early stages of childhood obesity of metabolic profiles potentially predicting obesity-related co-morbidities later in life should be considered not only a research topic, but also a clinical priority^4^. Besides the well-known biochemical and anthropometric risk factors present in overweight/obese children and associated to metabolic/cardiovascular disturbances in adulthood, new players are emerging as potential markers of these conditions.

A wide array of volatile organic compounds (VOCs) is emanated from the human body via breath, saliva, blood, milk, skin secretions, urine, and faeces, as products of metabolic processes^5^. Several studies have revealed that the metabolomics analysis of VOCs from biological fluids can give useful information for the clinical diagnosis and the therapeutic monitoring of a variety of pathologies, including gastrointestinal disorders and cancer^6,7^. In particular, Alkhouri *et al*. have provided evidence on significant differences of the pattern of exhaled VOCs in obese children compared with lean controls, demonstrating that various breath VOCs could potentially be useful to gain insight into pathophysiological processes and pathways leading to the development of childhood obesity and its related complications^8^.

Among the various biological fluids, urine shows specific features that make it an option of choice for volatile metabolomic profiling. Urine samples can be easily and non-invasively collected in large quantities and stored for long periods. They also offer higher concentrations of VOCs compared to other body fluids. A large body of evidence has revealed that urinary VOCs profiles contain rich information about individual physiological conditions, so that some urinary VOCs can be considered potential biomarkers in diagnosing or monitoring several pathological conditions, including diabetes, autism syndrome and different types of cancer^6,9^. Very recently, Elliot *et al*. have examined, by proton (1 H) nuclear magnetic resonance (NMR) spectroscopy and ion exchange chromatography, urinary metabolites from urine samples collected over two 24-hour time periods, to characterize the metabolic patterns of adiposity in a large epidemiological study in the United States and UK. This study showed unforeseen dependencies and interconnectivities between specific urinary metabolites and biochemical pathways that are possibly involved in the pathogenesis of obesity^10^.

The potential of urinary VOCs profiling as early diagnostic method has not been fully explored, both because of its complexity (containing numerous volatile compounds with different structure and a range of polarity, concentration and volatility) and of the analytical difficulties in identifying and quantifying volatile metabolites. Consequently, several analytical techniques have been developed for separation and concentration of VOCs from this biological fluid. Among them, solid-phase microextraction (SPME), a pre-concentration technology, which integrates sampling, extraction, concentration, and sample introduction into a single solvent-free step^11^, can be successfully used to simplify this complexity, specifically when coupled to capillary gas chromatography–mass spectrometry (GC-MS)^12^. Nowadays, urine analysis by SPME GC-MS has been well established as an easy, fast and reliable diagnostic tool allowing the identification of possible urinary disease-associated VOCs.

Aim of the present study was to evaluate, using SPME GC-MS, urinary metabolic signatures in a sample of normal-weight (NW) and overweight/obese (OW/Ob) children belonging to the Italian cohort of the I. Family study.

## Results

Twenty-one OW/Ob (ten females and eleven males, age 12.4 ± 1.2 years, BMI 26.7 ± 4.2 kg/m^2^) and twenty-eight NW (sixteen females and twelve males, age 12.9 ± 1.5 years, BMI 19.5 ± 1.8 kg/m^2^) children were included in the study. The total energy intake, and the energy intake (% kcal) from fat, carbohydrates and proteins were comparable in the two groups (Table 1). Similarly, no difference were observed between the two groups with regard to blood glucose, insulin, HbA1C, and HOMA index.

**Table 1:** Characteristic of the study population.

	**Nw**	**Ow/Ob**	**P-value**
N (m/f)	28 (12/16)	21 (11/10)	
Age (years)	12.9 ± 1.5	12.5 ± 1.1	0.288
BMI (Kg/m^2^)	19.5 ± 1.8	26.7 ± 4.2	<0.001
Height (cm)	155.0 ± 8.8	153.7 ± 8.2	0.364
Weigth (Kg)	47.2 ± 7.8	62.7 ± 13.9	<0.001
Glucose (mg/dl)	93.8 ± 5.9	94.1 ± 6.2	0.879
HOMA-IR	1.8 ± 1.2	1.9 ± 1.4	0.794
Energy (Kcal/day)	1715.9 ± 678.7	1748.0 ± 889.3	0.889
Energy fat (%)	32.0 ± 7.6	27.7 ± 7.4	0.057
Energy carbohydrate (%)	51.2 ± 8.3	55.4 ± 10.5	0.138
Energy protein (%)	16.3 ± 3.9	15.7 ± 6.1	0.658

M ± SD.

### Characterization of the volatile urinary metabolome under acidic and alkaline conditions

#### Determination of the urinary volatile profiles of overweight/obese and normal-weight children by SPME GC-MS

**Figure 1:**
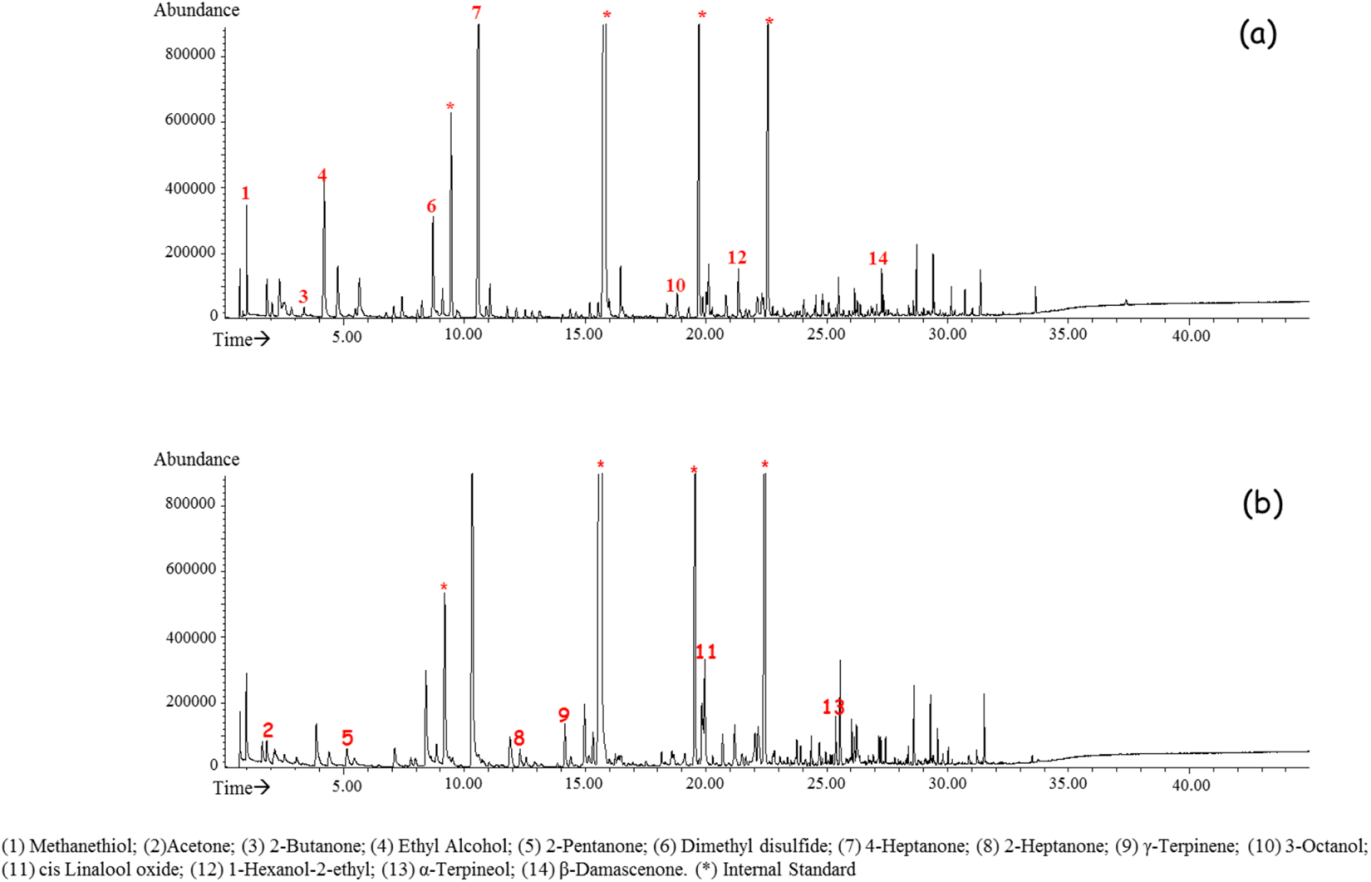
Representative SPME GC-MS chromatograms of urine VOCs from a NW (**a**) and OW/Ob (**b**) child obtained under acidic pH.

Typical SPME GC-MS TIC chromatograms of urine samples from a NW and an OW/Ob child, reported, respectively, in Fig. 1a and b, show that very similar VOCs profiles were obtained from the urine of the two groups of subjects, when analysed under acid conditions.

**Figure 2:**
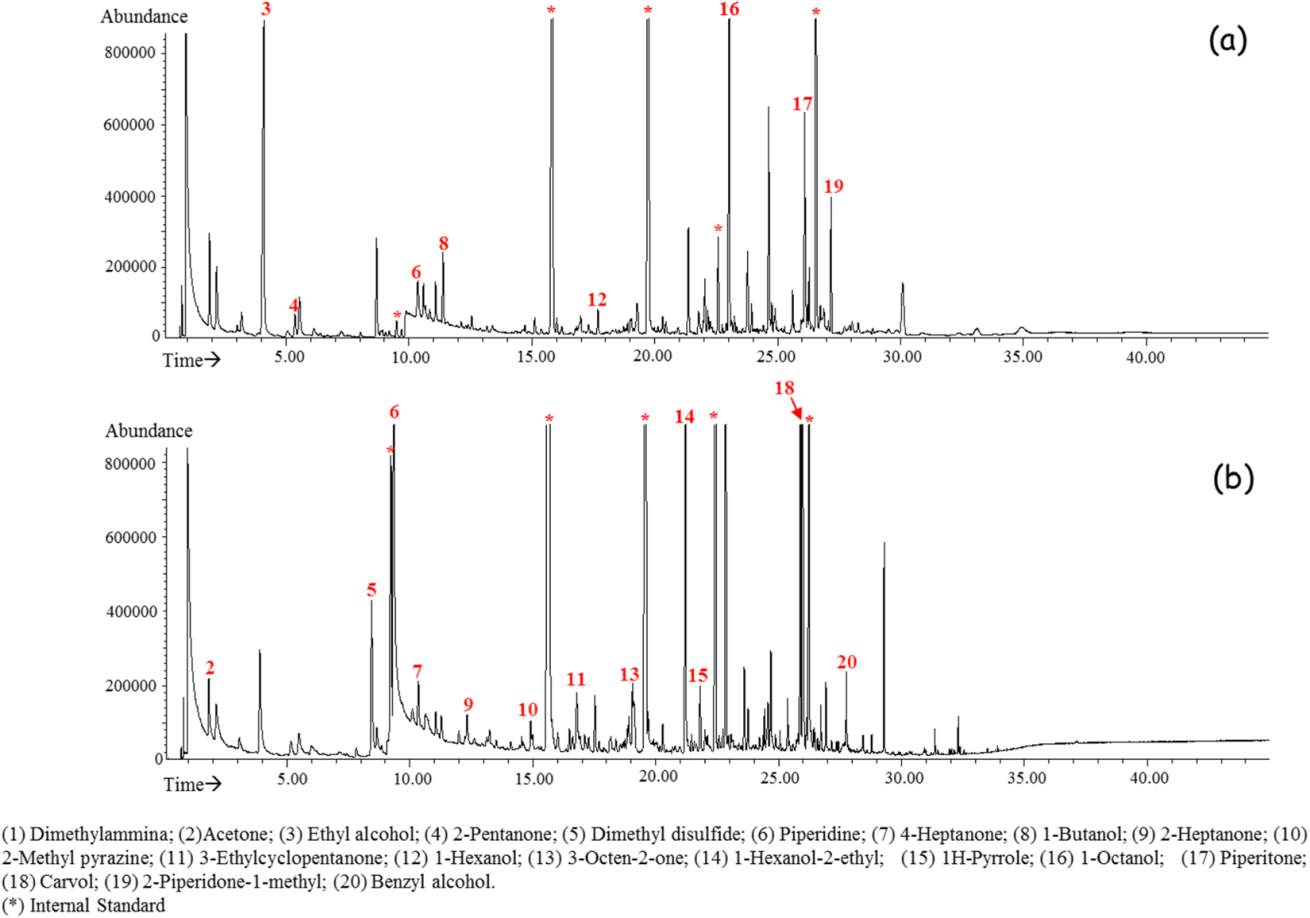
Representative SPME GC-MS TIC chromatograms of urine VOCs from a NW (**a**) and OW/Ob (**b**) child obtained under alkaline pH.

On the other hand, different volatile profiles were clearly distinguished in the urine obtained from the two groups of subjects analysed under basic pH, as displayed in Fig. 2a and b, which show, respectively, representative SPME GC-MS TIC chromatograms of urine samples from a NW and an OW/Ob child analysed under alkaline conditions.

Identification of each metabolite was achieved by comparing the fragmentation patterns (in terms of presence and intensity of the signals) with those in the NIST 2005 and Wiley 2007 libraries and by evaluating their retention times, using an in-house retention-time library based on reference standard samples. Additionally, identification of volatile compounds was also accomplished by matching their retention indices (RI) (as Kovats indices)^13^ with literature data, calculated in relation to the retention time of a C_8_-C_20_ n-alkanes series, with those of authentic compounds or literature data for similar chromatographic columns.

The identified metabolites included a variety of chemical structures: aldehydes, ketones, nitrogen compounds, terpenes, acids, alcohols, benzene derivatives, furan and sulphur-containing compounds and esters.

One hundred and ten and eighty-three metabolites were detected in samples from both NW and OW/Ob children under acid and alkaline conditions, respectively.

The fragment ion m/z values of the all identified urinary VOCs with the highest abundance within each fragmentation pattern, the matching percentage of the NIST and/or Wiley library, the experimental and literature reported Kovats index, the identification methods and their frequency of occurrence in OW/Ob and NW children are listed in Tables 2 and 3 for acidic and alkaline conditions, respectively.

**Table 2:** VOCs identified in the urine of OW/Ob and NW children under acid pH. Main fragment ion m/z, match percentage to the NIST 05 and/or Wiley 07 libraries, experimental (RIcal) and literature reported (RI) Kovats index, identification methods (ID) and percentage of occurrence are reported.

Metabolites	m/z	Match per cent (%)	RIcal	RI	ID	Percentage of occurrence (%)	
						NW	OW/Ob
Ketones							
Acetone	43	80			MS/S	100	100
2-Butanone	43	90			MS/S	93	91
2-Pentanone	43	72			MS/S	100	100
3-Pentanone 2,4-dimethyl	43	64			MS	30	4
2-Pentanone 4-methyl	43	53	1008	1008	RI/MS	59	65
2-Pentanone-3-methyl	43	60	1011	1013	RI/MS	59	65
3-Hexanone	43	91	1057	1057	RI/MS	100	96
4-Heptanone	43/71	91	1134	1131	RI/MS	100	100
3-Heptanone	43/72	64	1160	1162	RI/MS	18	17
2-Heptanone	58	87	1192	1191	RI/MS	96	96
4-Octanone	43	90	1235	1236	RI/MS	33	39
6-Methyl-5-hepten-2-one	43	87	1349	1347	RI/MS	30	30
2-Nonanone	58	68	1395	1395	RI/MS	81	78
Isophorone	82	86	1402	1607	RI/MS	44	39
Pinocarvone	81/108	76	1577	1585	RI/MS	93	100
p-Menthone	112	98	1504	1478	RI/MS	4	0
4-Methylacetophenone	119	94			MS	4	9
Alcohols							
2-Propanol	45	80			MS/S	4	
Ethanol	45	72			MS/S	100	100
3-Buten-2-ol 2-methyl	71	87	1048	1048	RI/MS	41	56
Isoamyl alcohol	55	78	1223	1222	RI/MS	15	4
1-Hexanol	56	50	1359	1358	RI/MS	74	65
3-Octanol	59	83	1402	1401	RI/MS	41	48
1-Heptanol	70	72	1455	1454	RI/MS	59	52
1-Hexanol-2-ethyl	57	86	1504	1503	RI/MS	96	87
1-Octanol	56	80	1569	1566	RI/MS/S	89	87
Isopulegol	41	95	1573	1574	RI/MS	26	9
Endo fenchol	81	94	1586	1579	RI/MS/S	33	13
2-Furanmethanol	98	95	1674	1678	RI/MS	89	91
1-Decanol	55	83	1776	1778	RI/MS	7	9
Nerol	69	91	1795	1794	RI/MS/S	—	4
Geraniol	69	81			MS/S	11	9
Aldehydes							
2-Methyl butanal	57	59			MS/S	93	91
3-Methyl butanal	44	64			MS/S	81	78
Pentanal	44	90			MS/S	4	22
Hexanal	44	94	1088	1087	RI/MS	100	100
Heptanal	70	93	1196	1195	RI/MS	41	52
2-Hexanal (E)	55	93	1231	1230	RI/MS/S	44	22
Furfural	96	78	1475	1474	RI/MS	37	48
Myrtenal	79	95	1642	1642	RI/MS	74	74
Benzaldehyde	105	90	1537	1537	RI/MS/S	15	—
Phellandral	109	95	1738	1741	RI/MS	11	22
Acids							
Acetic acid	43	80	1463	1465	RI/MS	67	52
Propanoic acid 2,2-dimethyl	57	72	1586	1582	RI/MS	33	22
Nonanoic acid	60	94			MS/S	33	30
Terpenes							
α-Pinene	93	96	1020	1027	RI/MS/S	11	—
α-Fenchene	93	72	1054	1071	RI/MS/S	33	43
Camphene	93	92	1053	1053	RI/MS	4	4
Verbenene	91	70	1122	1126	RI/MS	74	78
Phellandrene	93	90	1168	1177	RI/MS	59	61
β-Myrcene	93	83	1171	1171	RI/MS/S	15	17
α- Terpinene	121	97	1183	1183	RI/MS/S	93	87
1,5,8 p-menthatriene	91	94	1202	1210	RI/MS	41	30
dl-limonene	68	97	1205	1206	RI/MS/S	74	91
Eucalyptol	93	96	1215	1215	RI/MS	52	52
cis β-ocymene	93	74	1251	1250	RI/MS/S	41	30
γ- Terpinene	93	96	1258	1257	RI/MS/S	96	100
trans β-ocymene	93	89	1264	1250	RI/MS/S	30	30
p-cymene	119	97	1284	1282	RI/MS	100	100
m-cymene	119	94	1290	1282	RI/MS	100	96
α-Terpinolene	93/121	98	1297	1297	RI/MS/S	96	91
Tetrahydro linalool	73	59	1439	1431	RI/MS/S	85	78
cis linalool oxide	59	80	1451	1451	RI/MS/S	85	87
Dihydro myrcenol	59	80	1480	1473	RI/MS	89	83
trans-linalool oxide	59	91	1479	1483	RI/MS/S	78	61
Neroloxide	68	70	1484	1481	RI/MS	4	4
cis-theaspirane	138	93	1516	1507	RI/MS	100	96
Camphor	95	91	1529	1529	RI/MS/S	11	22
Vitispirane	192	95	1545	1543	RI/MS	100	100
Linalool l	71	97	1557	1558	RI/MS/S	85	83
1-Terpineol	81	96	1582	1581	RI/MS	52	65
4-Terpineol	71	95	1610	1616	RI/MS/S	93	87
γ-Valerolactone	56	60	1616	1617	RI/MS	15	22
Hotrienol	71	78	1616	1616	RI/MS	—	9
β-Cyclocitral	152	94	1629	1623	RI/MS	44	52
β-Terpineol	71	97	1629	1629	RI/MS	15	17
Menthol	71/81/95	93	1654	1652	RI/MS	85	83
Safranal	107	95	1661	1648	RI/MS	7	26
β-Ocimenol (Z)	93	86	1661	1627	RI/MS	7	22
trans-pinocarveol	92	90	1667	1661	RI/MS	89	91
α-Phellandren-8-ol	94	72	1680	1680	RI/MS	85	83
Borneol	95	80	1690	1688	RI/MS/S	11	26
Ocimenol	93	90	1712	1710	RI/MS	44	48
α- Terpineol	59	91	1667	1677	RI/MS/S	89	78
γ- Caprolactone	85	87	1719	1720	RI/MS	7	4
β- Phellandren-8-ol	94	80	1744	1778	RI/MS	74	65
Carvone	82	97	1757	1751	RI/MS/S	52	39
Naphtalene 1,2-dihydro-1,1,6-trimethyl	157	97	1763	1751	RI/MS	89	87
α-Bisabolene	93	72	1783	1778	RI/MS		9
Myrtenol	79	96	1795	1796	RI/MS	74	78
δ-Caprolactone	42	87			MS/S	78	56
Cadinene	161	89			MS	15	9
β-Damascenone	69	98			MS/S	100	100
Carveol	109	70			MS/S	30	22
p-Cymen-8-ol	135	90			MS	81	87
Calacorene	157	93			MS	89	74
Nerolidol	69	91			MS/S	74	78
Furans							
Furan	68	87			MS/S	100	100
2-Methyl furan	82	70			MS/S	100	96
2,5-Dimethyl furan	96	97			MS/S	100	100
2,3,5-Trimethyl furan	110	64	1063	1063	RI/MS	96	87
2-Pentyl furan	81	90	1248	1243	RI/MS	93	91
Thiols							
Methanethiol	47	91			MS	100	100
Thiophene 2-methylthio	130	81	1541	1543	RI/MS	37	26
Others							
Methyl ethyl sulfide	61	90			MS	4	4
Dimethyl disulfide	94	97	1079	1071	RI/MS	100	100
Dimethyl trisulfide	126	97	1388	1384	RI/MS	100	91
Benzene 1,2,3-trimethyl	105	93	1346	1332	RI/MS	74	74
Heptanenitrile	82	87	1402	1396	RI/MS	11	
4-Acetyl-1-methylcyclohexene	95	90	1565	1568	RI/MS	15	26

**Table 3:** VOCs identified in the urine of OW/Ob and NW children under alkaline pH. Main fragment ion m/z, match percentage to the NIST 05 and/or Wiley 07 libraries, experimental (RIcal) and literature reported (RI) Kovats index, identification methods (ID) and percentage of occurrence are reported.

Metabolites	m/z	Match per cent (%)	RIcal	RI	ID	Percentage of occurrence (%)	
						NW	OW/Ob
**Ketones**							
Acetone	43	80			MS/S	100	100
2-Butanone	43	64			MS/S	93	83
2-Pentanone	43	72			MS/S	96	100
4-Methyl-2-pentanone	43	72	1005	1008	RI/MS	22	30
3-Hexanone	43	82	1057	1057	RI/MS	70	74
5-Methyl-3-hexanone	57	64	1082	1068	RI/MS	66	78
2-Hexanone	43	74	1088	1088	RI/MS	30	43
4-Heptanone	43	90	1137	1131	RI/MS	89	96
3-Penten-2-one	69	72	1140	1138	RI/MS	22	17
3-Penten-2-one-4-methyl	83	64	1143	1139	RI/MS	44	43
2-Methyl-4-heptanone	57	76	1161	—	MS	18	22
3-Heptanone	57	60	1165	1162	RI/MS	18	30
2-Heptanone	43	91	1196	1198	RI/MS	100	100
4-methyl-2-heptanone	43	80	1222	1224	RI/MS	55	61
4-Octanone	57	87	1238	1236	RI/MS	4	26
3-Octanone	57	90	1270	1272	RI/MS	52	56
3-Hepten-2-one	55	90	1316	1274	RI/MS	37	48
3-Methylcyclohexanone	69	64	1336	1333	RI/MS	26	30
3-Ethylcyclopentanone	83	64	1342	—	MS	37	43
6-Methyl-5-hepten-2-one	43	78	1349	1347	RI/MS	96	96
2-Cyclopenten-1-one 2-methyl	96	72	1378	1373	RI/MS	4	13
3-Octen-2-one	55	81	1414	1414	RI/MS	48	61
Pinocarvone	81	87	1577	1575	RI/MS	96	96
Seudenone	82	83	1597	1592	RI/MS	100	96
Pulegone	81	91	1660	1662	RI/MS	65	61
Acetophenone	105	87	1666	1669	RI/MS	48	56
Piperitone	82	94	1749	1748	RI/MS	96	74
Methylacetophenone	119	64	1788	1793	RI/MS	15	26
**Alcohols**							
Ethanol	45	80			MS/S	100	100
Isobutyl alcohol	43	64	1106	1107	RI/MS	15	9
1-Butanol	56	86	1159	1158	RI/MS	93	100
2-Methyl −4-pentanol	45	64	1180	1181	RI/MS	22	43
2-hexanol	45	72	1180	1179	RI/MS	22	35
3-Buten-1-ol- 3-methyl	56	80	1264	1264	RI/MS	44	52
1-Pentanol	42	78	1267	1270	RI/MS	93	91
1-Hexanol	56	72	1362	1358	RI/MS	100	100
Cyclohexanol	57	62	1406	1407	RI/MS	30	26
2,4,4-Trimethyl-1-pentanol	57	83	1402	1326	RI/MS	100	96
1-Octen-3-ol	57	50	1450	1450	RI/MS	11	22
1-Heptanol	70	86	1463	1463	RI/MS	89	87
1-Hexanol-2-ethyl	57	80	1503	1503	RI/MS	100	100
1-Octanol	56	91	1565	1566	RI/MS/S	100	100
D-Fenchyl alcohol	81	90	1589	1588	RI/MS	55	56
Benzyl alcohol	79	95			MS/S	100	100
**Nitrogen compounds**							
Trimethylamine	58	90			MS/S	96	96
Dimethylamine	44	86			MS/S	100	100
Isoxazole	69	43			MS	59	65
Piperidine	84	91	1116	1115	RI/MS	93	96
Pyridine	79	76	1192	1193	RI/MS	48	35
2,6-Dimethyl pyridine	107	91	1261	1266	RI/MS	37	35
2-Methyl pyrazine	94	90	1277	1274	RI/MS	100	100
2,5-Dimethyl pyrazine	108	87	1333	1332	RI/MS	59	48
Formammide N,N-dimethyl	73	64	1338	1328	RI/MS	4	17
2,6-Dimethyl pyrazine	108	87	1339	1338	RI/MS	63	61
Ethyl pyrazine	107	87	1346	1344	RI/MS	44	52
2,3-Dimethyl pyrazine	108	76	1355	1355	RI/MS	78	61
2,4,6-Trimethyl pyridine	121	91	1375	1378	RI/MS	30	43
Trimethyl pyrazine	122	74	1410	1410	RI/MS	44	22
Pyrazine 2-ethyl-6-methyl	121	87	1391	1390	RI/MS	89	91
Ethenyl pyrazine	106	90	1442	1438	RI/MS	0	9
1 H Pyrrole	67	87	1524	1524	RI/MS	100	96
1 H Pyrrole 2-methyl	80	86	1585	1580	RI/MS	78	56
Pyrrole 4-ethyl-2-methyl	94	78	1730	—	MS	85	87
Formammide N,N-dibutyl	72	94	1785	1773	RI/MS	81	83
1-Methyl-2-piperidone	113	60			MS	93	96
1-Piperidinecarboxyaldehyde	113	93	1781	1786	RI/MS	22	26
**Terpenes**							
4-Terpineol	71	97	1608	1616	RI/MS/S	93	91
Menthol	71	64	1653	1652	RI/MS	85	74
trans Pinocarveol	92	64	1672	1661	RI/MS	93	91
α-Terpineol	59	91	1717	1710	RI/MS/S	93	87
Borneol	95	60	1724	1723	RI/MS/S	74	91
Carvol	82	95	1756	1751	RI/MS/S	81	65
Myrtenol	79	96	1795	1796	RI/MS	100	91
**Furans**							
2,5-Dimethyl furan	96	90			MS/S	11	4
3-Acetoamidofuran	83	72	1369	—	MS	55	56
2-Acetylfuran	95	76	1516	1512	RI/MS	74	74
**Esters**							
2-Butenoic acid ethyl ester	69	80	1165		RI/MS	11	22
2-Hexenoic acid ethyl ester	97	97	1352	1357	RI/MS	0	9
Octanoic acid-2-methyl ethyl ester	102	72	1385	—	MS/S	0	4
**Compounds contain sulfur**							
Dimethyl disulfide	94	96	1076	1071	RI/MS	96	96
Disulfide methyl-2-propenyl	120	72	1297	1296	RI/MS	44	52
Trisulfide dimethyl	126	91	1388	1389	RI/MS	66	74
Dimethyl sulfone	79	62			MS	70	78

#### Data analysis of the SPME GC-MS data sets

A preliminary exploratory data analysis was performed using PCA, excluding the presence of outliers in both the data sets on the basis of the DModX test and the Hotelling’s T2 test (at level of 95%).

Statistical data analysis based on multivariate and univariate approaches performed on VOCs profiles obtained under acid pH did not show differences between the two analysed groups. The PLS-DA model built, considering the whole data set, did not pass the permutation test on the class response and the distribution of the AUC ROC in prediction, obtained during stability selection, showed median equal to 0.63 and 5^th^ percentile equal to 0.47. In addition, the minimum p-value of the t-test for the measured variables resulted to be 0.12 and the behaviour of the related ROC curves unsatisfactory.

**Figure 3:**
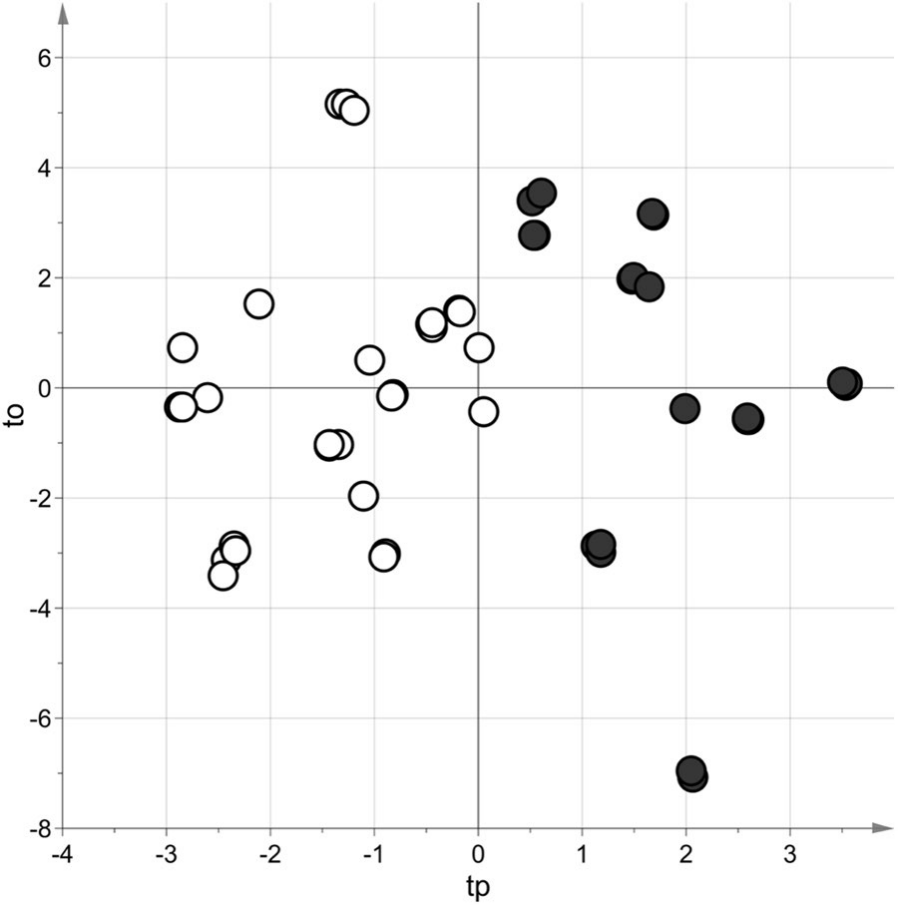
Score scatter plot of the PLS-DA model built considering the data set obtained under alkaline conditions. The model showed 2 components, R^2^ = 0.74 (p-value < 0.001) and AUC ROC, calculated by 7-fold cross-validation, equal to 0.96 (p-value < 0.001). NW children are indicated with white circles whereas OW/Ob subjects with dark grey circles. The PLS-DA model was post-transformed according to Stocchero & Paris (2016)^34^.

On the other hand, a robust PLS-DA model was obtained considering the SPME GC-MS data acquired in alkaline conditions. In Fig. 3 we report the score scatter plot of the discriminant model. Under stability selection, the distribution of the AUC ROC in prediction showed median equal to 0.91 and 5^th^ percentile equal to 0.64. The metabolites selected by stability selection were joined to those selected by t-test with False Discovery Rate and ROC obtaining a set of 14 putative markers, which seem to be crucial in the distinction of OW/Ob children and NW (Table 4). Among these, the levels of 2-pentanone, 3-hexanone, 5-methyl-3-hexanone, 4-methyl-2-heptanone, 3-octanone, 2,4,4-trimethyl-1-pentanol, 1-hexanol, 2-hexanol, 1-heptanol, dimethyl sulfone, 2,4,6-trimethyl-pyridine and formamide N,N-dibutyl are higher in the urine of OW/Ob children than in NW. In contrast, 1 H pyrrole-2-methyl and 1-methyl-2-piperidone have a lower concentration in OW/Ob children compared to NW.

**Table 4:** Selected VOCs identified in SPME GC-MS analysis under alkaline conditions.

ID	type^*^	AUC ROC (95% CI)^**^	power ROC^§^	spec^‡^	sens^+^	t-test p-value	power t-test^†^	q-value
5-Methyl-3-hexanone	OW/Ob > NW	0.606–0.899	0.89	1.00	0.52	2.5E-04	0.92	9.5E-03
1-Heptanol	OW/Ob > NW	0.563–0.879	0.79	0.79	0.71	3.4E-03	0.78	3.6E-02
4-Methyl-2-heptanone	OW/Ob > NW	0.543–0.840	0.66	0.64	0.71	3.8E-03	0.77	3.6E-02
2-Hexanol	OW/Ob > NW	0.541–0.815	0.60	0.79	0.52	6.4E-03	0.68	4.0E-02
Dimethyl sulfone	OW/Ob > NW	0.548–0.852	0.70	0.71	0.71	7.5E-03	0.72	4.0E-02
Formammide N,N-dibutyl	OW/Ob > NW	0.515–0.828	0.56	0.61	0.67	1.6E-02	0.56	5.0E-02
1-Hexanol	OW/Ob > NW	0.448–0.782	0.29	0.71	0.57	2.1E-02	0.51	6.0E-02
2-Pentanone	OW/Ob > NW	0.556–0.881	0.78	0.82	0.67	2.2E-02	0.49	6.0E-02
2,4,6-Trimethyl-pyridine	OW/Ob > NW	0.508–0.804	0.49	0.68	0.62	2.7E-02	0.46	6.5E-02
3-Hexanone	OW/Ob > NW	0.517–0.829	0.57	0.64	0.71	2.8E-02	0.49	6.5E-02
3-Octanone	OW/Ob > NW	0.637–0.903	0.93	0.75	0.81	5.0E-02	0.50	8.2E-02
2,4,4-Trimethyl-1-pentanol	OW/Ob > NW	0.529–0.837	0.62	0.54	1.00	7.6E-02	0.32	1.1E-01
1 H pyrrole-2-methyl	NW > OW/Ob	0.588–0.862	0.80	0.61	0.90	1.3E-03	0.96	2.4E-02
1-Methyl-2-piperidone	NW > OW/Ob	0.526–0.831	0.60	0.75	0.52	1.3E-02	0.77	4.9E-02

^*^OW/Ob > NW indicates metabolites with concentration higher in the urine of OW/Ob children than in NW; NW > OW/Ob indicates metabolites with lower concentration in OW/Ob children than in NW.

^**^AUC ROC (95% CI) = confidence interval of the Area Under the ROC curve at the level of 95%.

^§^power ROC = power for identifying the observed AUC given a level of significance α = 0.05.

^‡^spec = specificity.

^+^sens = sensitivity.

^†^power t-test = power for identifying the observed difference in the means given a level of significance α = 0.05.

## Discussion

In the present paper, SPME GC-MS was used to evaluate for the first time the volatile urinary metabolic signatures associated with early obesity on a sample of children belonging to the Italian cohort of the I. Family study.

Urine sampling is a simple and safe alternative to more invasive investigations in children, and, as far as we are aware, this is the first study in which, in order to profile a wider range of urinary volatiles with different physicochemical properties, VOCs from urine samples of OW/Ob and NW children have been analysed under both acidic and alkaline conditions.

One hundred and ten and eighty-three VOCs were detected in samples from both NW and OW/Ob children under acid and alkaline conditions, respectively. Statistical data analysis based on multivariate and univariate approaches performed on volatiles profiles obtained under acid pH did not allow distinguishing the two analysed groups. On the other hand, a robust PLS-DA model was gained considering the large and heterogeneous SPME GC-MS set of data acquired under alkaline conditions, which also allowed the identification of fourteen VOCs putative biomarkers that seem to be crucial in differentiating OW/Ob children from NW.

A large number of VOCs in urine seems to arise from the bacterial action in the gut^5^, while the presence of volatile metabolites in the gastrointestinal tract is believed to result from the complex interaction of colonocytes, human gut microflora and invading pathogens^14^. Alkhouri *et al*. have reported that obese children have a unique pattern of VOCs compared with lean children showing that obesity, like other pathological disturbances, can induce the synthesis of new VOCs or a modification in the concentration of VOCs that are normally produced into the metabolic condition of an individual^8^.

Specifically, alterations found in the pattern of VOCs can be reflective of changes and variations within the gastrointestinal environment, as demonstrated by a large body of evidence for the role of gut microbial dysbiosis in the pathophysiology of obesity and other gastrointestinal disorders^15^.

The relationship between the intestinal microbiota and the immune system of the host could be a mediating factor in the development of obesity^16^. Indeed, there is evidence that the gut microbiota can directly influence body weight in several ways^17^. The relative abundance of bacterial species and the microbial diversity vary with the physiological state of the host. In particular, obesity is associated with both a reduced-diversity microbial community and an altered representation of bacterial genes^15^.

Microbes produce about 300 volatile organic compounds in the human gut, whose systemic effects are unknown^18^. Del Chierico *et al*. (2017) have demonstrated that the relative abundance of some species of bacteria (*Firmicutes* and *Bacteroidetes*) in obese children was similar to that of nonalcoholic fatty liver (NAFL) and nonalcoholic steatohepatitis (NASH) children. These microorganisms always present higher levels in patients compared to controls, while the level of other type of bacteria (*Oscillospira*) is decreased. Consequently, VOCs have huge potential as biomarkers specific of gastrointestinal and even metabolic diseases.

Data from the literature suggest a possible biological role for some, but not all, of the fourteen VOCs whose levels significantly differed in the two groups under study.

The up-regulation of some of the ketonic and alcoholic compounds, reaching statistical significance in the OW/Ob group vs NW, has been already observed in obese compared to normal-weight children and explained with a gut microbial dysbiosis in the obese subjects^17^. In particular, 5-methyl-3-hexanone and 4-methyl-2-heptanone, belonging to the methyl-ketone group, were found statistically higher in OW/Ob than in NW children. These compounds can be produced by many species of bacteria and fungi from the respective alkanoic acid^19^. Indeed, the other ketonic compounds, such as 2-pentanone, 3-hexanone and 3-octanone, can also be synthesized by bacteria^5^.

With regard to alcohols, the abundance of 1-hexanol, 2-hexanol, 1-heptanol, 2,4,4-trimethyl-1-pentanol is increased in all OW/Ob children compared to NW. These findings can be explained with same results reported in Zhu *et al*. (2013), who have shown that there is a significant increase of Bacteroides in the obese and NASH groups, compared to the healthy group. The increase of these gut microbial bacteria, capable of producing alcohol, may explain why some alcoholic VOCs are more abundant in OW/Ob children compared to NW^20^.

A more in depth assessment of the literature has allowed to retrieve different papers indicating that some bacterial species, present also in the human gut microbiome, may produce some of the compounds we found. Specifically, 5-methyl-3-hexanone, 2-pentanone, 3-octanone and 1-hexanol can be released by various actinomycetes in gut flora^21–23^.

The VOCs profile of OW/Ob compared to NW children also results in a higher urinary level of dimethyl sulfone in OW/Ob children. Sulphur containing compounds are formed by incomplete metabolism of sulphur containing amino acids in the transamination pathway. The levels of these compounds are known to be elevated in patients with altered liver function^24^. Fatty liver disorders are very common in obese children and adolescents, reaching a prevalence of 40–50%^25^. Interestingly, previous findings have demonstrated that sulphur-containing compounds are also associated to childhood obesity^8^.

Our study confirms the results of recent papers indicating that certain urinary volatile compounds appeared to contribute to the metabolic signature of adiposity^8,10,17^.

The VOCs associated with obesity in our study are indeed not consistent with those identified in other studies^8,10^. Several factors may affect the different VOCs profiles observed in different settings, including environmental and dietary factors, methodological differences in sampling (urine/breath) and analytical detection techniques, and finally, characteristics of the populations under study. Of note, Elliot *et al*. (2015) reported about adult subjects, while in the Alkhouri *et al*. study (2015) a large proportion of affected children had severe obesity.

Moreover, although we did not report differences in caloric as well as macronutrient intake between OW/Ob children and NW, we cannot exclude those differences in specific dietary components may affect the VOCs profile. It has been suggested that both intra- and inter-variability in VOCs profile can be related to dietary habits^26^. Consequently, the adoption of a standardized diet prior to the test can help to reduce variability in VOCs in future experimentations. Finally, the present cross-sectional analysis, with urinary VOCs determined at a single time point, by its nature excluded the identification of causality.

The absence of a blind validation set to test our findings is a limitation of the study. In spite of the robust and conservative procedure used for internal validation, a new set of subjects would be required to replicate the results of this pilot study and to confirm the selected putative markers.

Post-hoc power analysis suggested that the number of recruited subjects for each group was insufficient for some of the selected VOCs. We will take into account our results for designing new experiments with a sufficient statistical power able to improve the knowledge about the role of the VOCs in explaining the development and the progression of the obesity in children.

Finally, environmental chemical exposures possibly interfering with the urinary VOCs profile were not assessed in the present study.

In conclusion, our results suggest that there is potential for urinary VOCs, detected by SPME GC-MS, as metabolic biomarkers of childhood obesity. In particular, the hypothesis that altered urinary VOCs profiles may reflect gut dysbiosis or early impairment of the liver function deserves further investigation, particularly considering that urine sampling represents simple and safe alternative to more invasive procedures in children. While we recognize the limitations and the relative reliability of our analyses, these novel findings may be considered as hypothesis-generating, to be obviously confirmed by larger prospective investigations.

## Methods

### Experimental design and cohort

The I.Family project (http://www.ifamilystudy.eu) aimed to assess the determinants of eating behaviour in children and adolescents of eight European countries and related health outcomes was built on the IDEFICS cohort (http://www.ideficsstudy.eu), established in 2006 and followed-up in 2012–2013. A full description of the project has been recently published^27^.

Briefly, the Italian cohort of the I.Family project was composed by 1521 children and teens (773 NW, 748 OW/Ob) who underwent a general examination module^27^. Among them, 249 participants (121 NW, 128 OW/Ob), identified on the basis of their body weight trajectories over the 6 year follow-up, underwent additional examinations, including the collection of a fasting urine sample. Among the 249 participants asked to provide an additional 50 ml fasting urine sample, a subsample of 28 NW and 21 OW/Ob participants accepted, and was included in the present analysis.

In particular, weight, to the nearest 0.1 kg with children wearing light clothes and without shoes was measured using an electronic scale (TanitaBC420SMA,Tanita Europe GmbH, Sindelfingen, Germany). Height was measured using a telescopic height-measuring instrument (Seca 225 stadiometer, Birmingham, UK) to the nearest 0.1 cm. BMI was calculated as weight (in kg) divided by height squared (in m^2^). A detailed description of the anthropometric measurements, including intra- and inter-observer reliability, has been previously published^28^. Weight categories were defined according to age- and sex-specific BMI categories^29^.

Each individual on the day of the physical examination provided a sample of morning urine (after overnight fasting) in a 50 mL sterile PVC container. Samples were immediately frozen and stored at −80 °C until analysis. The complete defrosting of the samples was performed at room temperature shortly before analysis. Dietary intake of the previous 24 h was assessed using an online 24-h dietary recall assessment program based on the validated offline version^30^.

Children were asked to participate, on voluntary basis, in fasting blood withdrawal. A detailed description of sample collection and analytical procedures has been published by Peplies *et al*. (2010)^31^.

Specifically, serum insulin was measured through enzyme-linked immunosorbent assay kit (MODULAR E170, Roche Diagnostics). Insulin resistance was estimated by the Homeostatic Model Assessment (HOMA-IR), using the following formula: HOMA-IR = [serum insulin (mU/L) × blood glucose (mmol/L)]/22.5^32^.

The study protocol was approved by the local Ethics Committee of the local Health Authority (ASL Avellino) and informed written parental consent was obtained for each participant. All experiments were performed in accordance with relevant guidelines and regulations.

### Chemicals and reagents

2-β-pinene (97% purity), 2-octanone (98% purity), 4-hexen-ol (96% purity), ethyl-nonanoate (98% purity), trans-2-decenal (92% purity), and aniline (98% purity) were used as internal standards, and were all produced by Sigma-Aldrich. Stock solution of these six standards, at a concentration of 1000 ppm, were prepared by dissolving the standards in a mixture of Mill-Q water and ethanol (95/5 (v/v)), and were stored in a refrigerator at 4 °C.

Ethanol was purchased from Romil. Ultra-pure water from a Milli-Q system (Millipore, Bedford, MA, USA) with a conductivity of 18 MΩ was used throughout.

Sodium chloride (NaCl), potassium carbonate (K_2_CO_3_) and potassium hydroxide (KOH) were from Sigma-Aldrich, and hydrogen chloride (HCl) was from Carlo Erba. Helium at a purity of 99.999% (Rivoira, Milan) was used as the GC carrier gas. The SPME fibers and the glass vials were purchased from Supelco (Bellofonte, PA, USA). The capillary GC-MS column HP-Innowax (30 m × 0.25 mm × 0.5μm) was obtained from Agilent J&W (Agilent Technologies Inc. Santa Clara, CA).

The SPME fibers were conditioned as suggested by the manufacturer, prior to their first use. Before the initially daily analysis, the fibers were conditioned for 5 min at the operating temperature of the GC injector port and the blank level was checked. Triplicate analyses were performed.

### Sample preparation and SPME procedure

Volatiles profiling was performed using the headspace SPME GC-MS method described by Cozzolino *et al*. (2014), with a DVB/CAR/PDMS (50/30 μm) fibre, an extraction temperature of 40 °C and an extraction time of 30 min.

The pH of urine samples can be an important aspect in affecting the extraction of VOCs. Although both ionized and un-ionized forms of acidic and basic VOCs exist in urine, only the un-ionized forms are volatile and can be found in the headspace. Consequently, in order to provide a profile that represents the true concentrations of VOC components in urine, here urine samples were analysed both under acid and alkaline pH, following two different sample preparation procedures, as shown below^9^.

1. Acid conditions (pH 1–2): in a 20 mL screw-on cap HS vial (Supelco, Bellefonte, PA, USA), 4 mL urine were added to 1 mL water, approximately 3 g NaCl and 100 μL 6 mol L^−1^ HCl;
2. Alkaline conditions (pH 12–14): 4 mL urine, 1 mL water, approximately 3 g K_2_CO_3_ and one pellet KOH were mixed in the HS vial.

In each sample12.5 μL from a stock solution of the six internal standards (2-β-pinene, 2-octanone, 4-hexen-ol, ethyl-nonanoate, trans-2-decenal, and aniline) at a concentration of 25 ppb were added.

After stirring, vials were sealed with a Teflon (PTFE) septum and an aluminium cap (Chromacol; Fisher, Loughborough, UK) for the release of volatile compounds in the vial and enable analysis.

The sample vial was placed in the instrument dry block-heater and held at 40 °C for 30 min to equilibrate the system. The extraction and injection processes were automatically performed using an autosampler MPS 2 (Gerstel, Mülheim, Germany). Finally, the fibre was automatically inserted through the vial’s septum for 10 min, to allow the volatiles adsorption onto the SPME fibre surface.

### Gas chromatography–mass spectrometry analysis

The SPME fibre was introduced into the injector port of the gas chromatograph (model 7890 A; Agilent Technologies, Santa Clara, CA) coupled with a mass spectrometer 5975 C (Agilent), wherein the metabolites were thermally desorbed and directly transferred to a capillary column HP-Innowax (30 m × 0.25 mm × 0.5 μm; Agilent) for analysis.

The oven temperature program was initially set at 35 °C for 5 min, ramped to 120 °C at 5 °C min^−1^, increased to 250 °C at 10 °C min^−1^, and held for 10 min. The temperature of the ion source and the quadrupole were held at 230 °C and 150 °C, respectively; helium was used as carrier gas with a flow of 1.5 mL min^−1^; injector temperature was kept at 240 °C and the pulsed splitless mode was used for the analysis.

The fibre was maintained in the injector for 25 min. Mass spectra were acquired at an ionization energy of 70 eV and volatile components were detected by mass selective detector. The detector operated in a mass range between m/z 30 and 300 with a scan rate of 2.7 scans/s. Each sample was analysed in triplicate in a randomized sequence where blanks, related to analyses of coating fibre not submitted to any extraction procedure, were run.

Metabolites identification was accomplished by searching mass spectra in the available database libraries (NIST, version 2005; Wiley, version 2007) and by the comparison of their retention times with an in-house developed retention time library based on commercial standards. Furthermore, identification of volatile compounds was also achieved by matching their retention indices (RI) (as Kovats indices; Kovats, 1958) with literature data, determined relative to the retention time of a C_8_-C_20_ n-alkanes series, with those of authentic compounds or literature data.

Metabolite concentration was determined by calculating the ratio of the peak area of the metabolite and the peak area of the related internal standard. After the calculation of the median of the triplicates, the obtained data sets were log-transformed and autoscaled.

### Statistical analysis

The collected data were investigated by multivariate and univariate statistical data analysis.

Specifically, exploratory data analysis was performed by Principal Component Analysis (PCA), whereas Projection to Latent Structures Discriminant Analysis based on Variable Influence on Projection selection (PLS-DA VIP-based) was applied to identify the differences between OW/Ob and Nw children. Stability selection based on Monte-Carlo sampling was used to highlight the subset of relevant variables characterizing the two groups and to estimate the predictive power of the models^33^. During stability selection, three hundred random subsamples of the collected samples were extracted by Monte-Carlo sampling (with a prior probability of 0.70), and then PLS-DA VIP-based was applied to each subsample, obtaining a set of 300 discriminant models. The predictive performance of each model was estimated by means of Receiver Operating Characteristic (ROC) curve analysis of the outcomes of the predictions of which samples would be excluded during sub-sampling. Within this set of PLS-DA VIP-based models, the most frequently selected variables were identified as relevant variables. The threshold of VIP to use for variable selection was determined maximizing the Q2 parameter (i.e. R2 calculated by cross-validation) during 7-fold cross-validation. Models were submitted to permutation test on the class response to avoid over-fitting according to good practice for model building.

ROC analysis and t-test with False Discovery Rate were applied to investigate the properties of single variable. We considered VOCs with t-test p-value less than 0.05, q-value less than 0.1 and AUC ROC greater than 0.50 (α = 0.05) as significant variables. The results of the multivariate data analysis were merged to those obtained by univariate data analysis to have a comprehensive data analysis, where both the correlation structures and the individual properties of the measured variables were taken into account.

PCA, PLS-DA VIP-based with stability selection, ROC analysis and t-test with False Discovery Rate were implemented with the R 3.1.2 platform (R Foundation for Statistical Computing).

### Data availability

The datasets generated during and/or analysed during the current study are not publicly available according to the conditions laid down in the Consortium Agreement of the I.Family project (EC FP7 Grant Agreement No. 266044) but are available from the corresponding author on reasonable request.
